# Solo-surgeon pure laparoscopic donor nephrectomy using passive camera holder: IDEAL stage 2a study

**DOI:** 10.1186/s12894-022-00996-8

**Published:** 2022-03-25

**Authors:** Dong Hyeon An, Jae Hyeon Han, Myoung Jin Jang, Joomin Aum, Yu Seon Kim, In Gab Jeong, Bumsik Hong, Dalsan You

**Affiliations:** 1 Department of Urology, Asan Medical Center, University of Ulsan College of Medicine, Seoul, Korea; 2 Department of Urology, Korea University Ansan Hospital, Korea University College of Medicine, Ansan, Korea; 3 Asan Institute for Life Sciences, Asan Medical Center, Seoul, Korea; 4 Department of Urology, Asan Medical Institute of Convergence Science and Technology, Asan Medical Center, University of Ulsan College of Medicine, 88, Olympic-ro 43-gil, Songpa-gu, Seoul, 05505 Korea

**Keywords:** Laparoscopy, Living donors, Solo practice, Camera holder, Feasibility, Complication

## Abstract

**Background:**

Solo-surgery can be defined as a practice of a surgeon operating alone using a camera holder, without other surgical members except for a scrub nurse. This study was designed to evaluate the feasibility and safety of solo-surgeon pure laparoscopic donor nephrectomy.

**Methods:**

The study protocol was approved by the Institutional Review Board of Asan Medical Center, Seoul, Korea. The brief study protocol was registered on the Clinical Research Information Service site of the Korea Centers for Disease Control and Prevention. Candidates fulfilling all inclusion and exclusion criteria were enrolled in the clinical trial and underwent solo-surgeon pure laparoscopic donor nephrectomy. The feasibility was assessed by the proportion of subjects who could undergo solo-surgeon pure laparoscopic donor nephrectomy without difficulty. The perioperative complications were identified to assess the safety of solo-surgeon pure laparoscopic donor nephrectomy.

**Results:**

Of the 47 potential candidates from November 2018 to August 2019, 40 were enrolled in the clinical trial and seven excluded due to declining participation. The feasibility of solo-surgeon pure laparoscopic donor nephrectomy was 100%, without an occasion of any difficulty requiring conversion to the human assisted pure laparoscopic donor nephrectomy. Fourteen intraoperative complications occurred in 10 patients. The most common intraoperative complication was spleen injury. Two of three cases classified as the Satava classification grade II were due to the incomplete stapling of endoscopic stapler. Seventy-eight postoperative complications occurred in 34 patients. The most common postoperative complication was nausea/vomiting and followed by aspartate aminotransferase/alanine aminotransferase elevation. Most postoperative complication was independent of the solo-surgery itself.

**Conclusions:**

Solo-surgeon pure laparoscopic donor nephrectomy using passive camera holder is technically feasible. In terms of safety, it is necessary to adjust the scope of surgery performed alone.

*Trial Registration* CRIS, KCT0003458. Registered 30/01/2019, Retrospectively registered, https://cris.nih.go.kr/cris/search/detailSearch.do/15868.

## Background

For stable surgical vision during laparoscopic surgery, the assistant who holds the camera should keep the image stable, focus the surgical site in the center of the monitor, and provide an unrotated image. However, since the human assistant is generally a resident, it is difficult to gather experience in maintaining a stable image. Even experienced assistants suffer from the difficulty of maintaining a stable image because of the development of fatigue. Human assistants holding a camera tend to have tremors and lose horizontal orientation. Moreover, there are frequent interruptions due to the need for cleaning of the lens after unintentional contact of the camera with the surrounding tissue [1]. The camera holder has been developed to overcome these problems and has the advantage of maintaining a stable camera position without any shaking. Through the use of a camera holder, a surgeon can perform laparoscopic surgery without the human assistant, called solo-surgery. Solo-surgery can be defined as a practice in which a surgeon operates alone, without other surgical staff members except for a scrub nurse.

Solo-surgery in the field of urology was first reported in 1995 by Partin et al. [2] and Kavoussi et al. [3]. They performed prostate surgery using a robotic system rather than a camera holding system. Solo-surgery in donor nephrectomy was first described in 2009 by Lee et al. [4] using passive camera holder in video-assisted minilaparotomy surgery.

Pure laparoscopic donor nephrectomy (PLDN) is technically tricky compared to radical or simple nephrectomy because donor nephrectomy requires short ischemic time and delicate dissection of renal vessels and ureter. Moreover, solo-surgery may take longer as the operator adjusts the camera position, and may take extra time for the medical personnel to join the surgery for assistance in case of an unexpected emergency. In addition, the fixed camera may interfere with the operation. Therefore, it is necessary to demonstrate the feasibility and safety of solo-surgeon PLDN (SS-PLDN). This Idea, Development, Exploration, Assessment, Long-term study (IDEAL) stage 2a study was designed to evaluate the feasibility and safety of SS-PLDN [5].

## Methods

### Study design and data collection

The study protocol was approved by the Institutional Review Board of Asan Medical Center, Seoul, Korea (No. 2018–0864), and the study was conducted in accordance with the Declaration of Helsinki and the International Conference on Harmonization Guidelines for Good Clinical Practice. The brief study protocol was registered on the Clinical Research Information Service site of the Korea Centers for Disease Control and Prevention (No. KCT0003458, Registered 30/01/2019). The potential candidates were living kidney donors, aged ≥ 18 and < 80 years who can speak in Korean language. The potential candidates provided written informed consent before participating in the clinical trial. Candidates were excluded if they were unable to undergo PLDN due to previous abdominal surgery; were unwilling to participate in the study; were inappropriate to participate in the study according to the investigator. Candidates fulfilling all inclusion and exclusion criteria were enrolled in the clinical trial and SS-PLDN was performed by a single surgeon (DY). The surgeon had 5 years of experience with PLDN before starting the present study and performed more than 200 of PLDN including 10 SS-PLDN.

The patients were assessed with respect to feasibility and perioperative complications of SS-PLDN. Additionally, baseline data, operative and convalescence parameters were prospectively collected. Baseline data included patient’s age, sex, height, body weight, medical and surgical histories, laterality, number of artery and vein, hemoglobin concentration, and glomerular filtration rate (GFR). GFR was estimated from serum creatinine concentration with a variation of the original Modification of Diet in Renal Disease formula [6].

### Surgical techniques

**Fig. 1:**
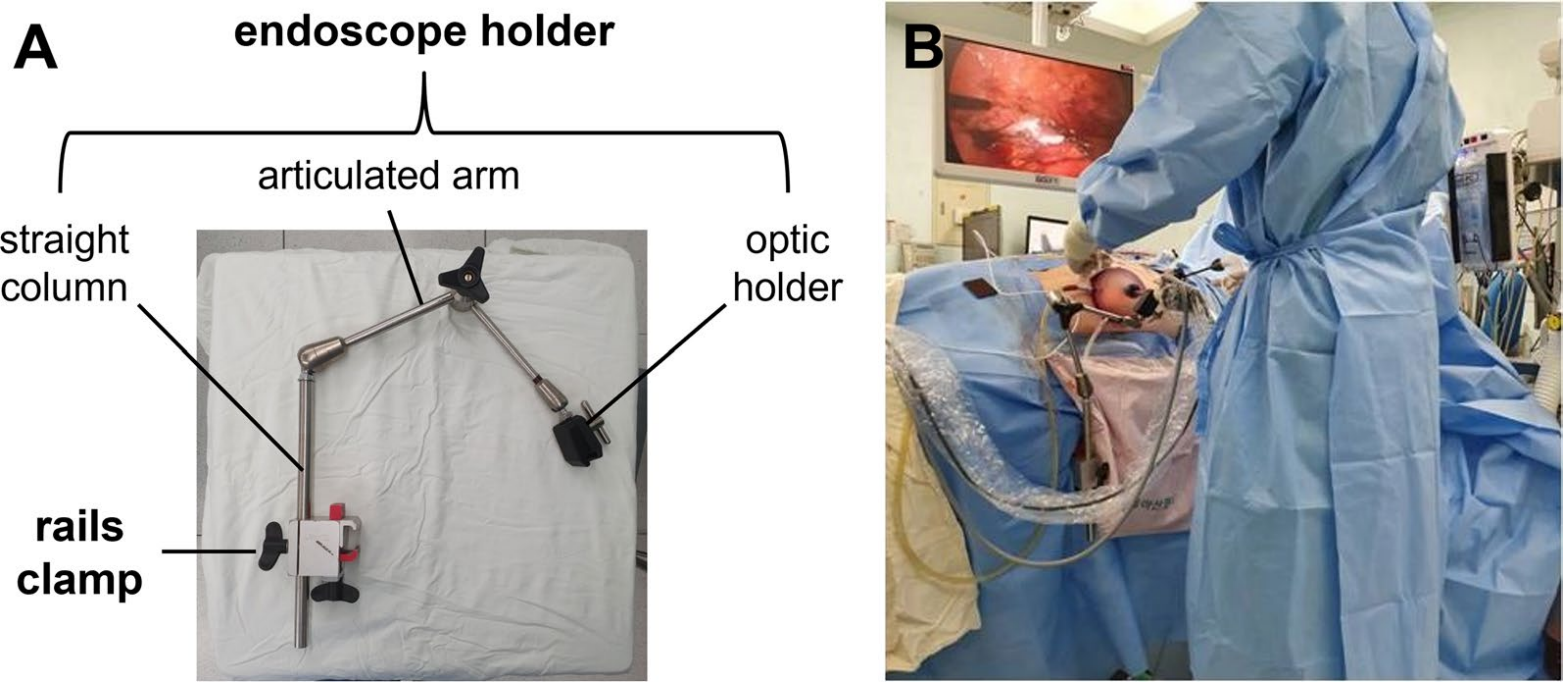
Passive camera holder (FISSO, Zurich, Switzerland). The camera holding system consists of endoscope holder and rails clamp, and the endoscope holder consists of optic holder, articulated arm and straight column (**A**). The camera holding system is used by securing the endoscope holder to the operating table with rails clamp (**B**)

SS-PLDN was implemented with some changes of the previously described PLDN [7, 8]. For right (left)-sided allografts, patients were placed in a 45–60° oblique position with the left (right) side down. A 6 cm omega-shaped incision was made around the umbilicus for insertion of a GelPOINT advanced access platform (Applied Medical, Rancho Santa Margarita, CA, USA). A 12 mm trocar was placed below the right (left) ribcage edge margin in the right (left) midclavicular line. Another 12 mm trocar was placed at the mid-level of umbilicus and anterior superior iliac spine in the right (left) anterior axillary line. A 5 mm trocar was placed just below the xiphoid process for right-sided allografts and used for liver retraction. The assistant holding the camera was replaced by the passive camera holder (FISSO, Zurich, Switzerland) (Fig. 1). This is confined from the completion of port placement until the completion of hemostasis. The white line of Toldt was cut to reflect the colon inward, and anterior renal fascia was entered near the renal hilum. The renal artery and vein were fully released from lymphatic and other perivascular tissues, taking care to avoid vascular damage. The ureter was dissected caudally to the level of the internal iliac vessels, leaving sufficient margins to ensure blood supply around it. After intravenous injection of heparin 5000 IU, a polymer clip was applied at the caudal end of the dissected ureter. The ureter was divided cephalad to the clip. The renal artery and vein were transected using an Endopath ETS-Flex articulating endoscopic linear stapler (Ethicon Endo-Surgery Inc., Cincinnati, OH, USA). The kidney was removed through the umbilical incision in a LapBag (Sejong Medical Co. Ltd., Paju, Korea), immediately placed in sterile ice slush, and delivered to the recipient team for transplantation. The scrub nurse assisted the surgeon during extracting the kidney. After the procurement, protamine sulfate 50 mg was administered intravenously. After reducing intraperitoneal pressure, the abdomen was carefully examined to control bleeding. Wound closure was done with an assistant.

### Primary outcomes

The feasibility was assessed by the proportion of subjects who could undergo SS-PLDN without difficulty. The difficulty may occur in three situations. First, if SS-PLDN is no longer possible owing to a collision between the camera holder with the camera and the surgeon's hand or laparoscopic instrument. Second, if there is a complication that requires the help of an assistant during solo-surgery. Third, when solo-surgery operation time (from the completion of port placement until the completion of hemostasis) exceeds 120 min. In all three situations, the operation is converted to the human-assisted PLDN. Evaluation criteria and reporting methods were the ratio of the subjects who underwent surgery as planned to all of those who planned to undergo SS-PLDN.

The perioperative complications were identified to assess the safety of SS-PLDN. The surgeon recorded all the intraoperative complications on the operation record and case report form. All operations were video-recorded and reviewed by two researchers (DHA & DY) to check for missing parts. Evaluation criteria and reporting methods follow the Satava classification of intraoperative complications [9]. Grade I represents an error without consequences. This may not be serious enough to cause complications. Grade II is defined as an error that can be resolved with minimal or no effect by immediate identification and correction. Grade III indicates an error accompanying the surgeon's apparent responsible consequence. Not only is an error committed, but it is also unrecognized.

The researchers collected all information that could be regarded as a postoperative complication for 90 days through vital signs, laboratory tests, and physical examination. The postoperative complications were classified into hematologic, infectious, gastrointestinal, procedural, vascular, metabolic, genitourinary, cardiac, respiratory, and others, and the severity was classified according to Clavien-Dindo classification of postoperative complications [10]. If postoperative complications occurred, the symptoms, start date, duration, intensity, and causal relationship of the complications were recorded. All postoperative complications were investigated until the end of clinical trial. Vital signs during hospitalization were measured at least three times daily and assessed for systolic and diastolic blood pressures, pulse rate, and body temperature. Laboratory test results were classified into normal and abnormal (based on clinical significance) according to the normal range of our institution. Physical examination included evaluation of the hematological system, infections, gastrointestinal tract, interventional procedures, cardiovascular system, metabolism, reproductive/urinary tract, respiratory system, and other examinations.

### Secondary outcomes

The operative parameters included total operation time, solo-surgery operation time, warm ischemia time, and estimated blood loss. Total operation time was defined as the time between skin incision for the umbilical incision and skin closure. Warm ischemia time was defined as the time from renal artery occlusion to immersion of the kidney in ice slush. The convalescence parameters included postoperative pain, interval to return to regular diet, hospital stay, postoperative hemoglobin, and postoperative GFR. Postoperative pain was assessed with a patient-reported visual analog scale.

### Statistical analysis

Considering that the study period was one year, 40 (80% of the approximately 50 annual donor nephrectomy performed) was decided as the target number of subjects for surgeons to perform SS-PLDN on. Quantitative data were expressed as mean ± standard deviation. Categorical data were expressed as frequency and ratio (%). The feasibility was analysed in intention-to-treat population. Perioperative complications and operative and convalescence parameters were analysed in patients who underwent SS-PLDN (actual-procedure-received population).

## Results

### Patients data

**Fig. 2:**
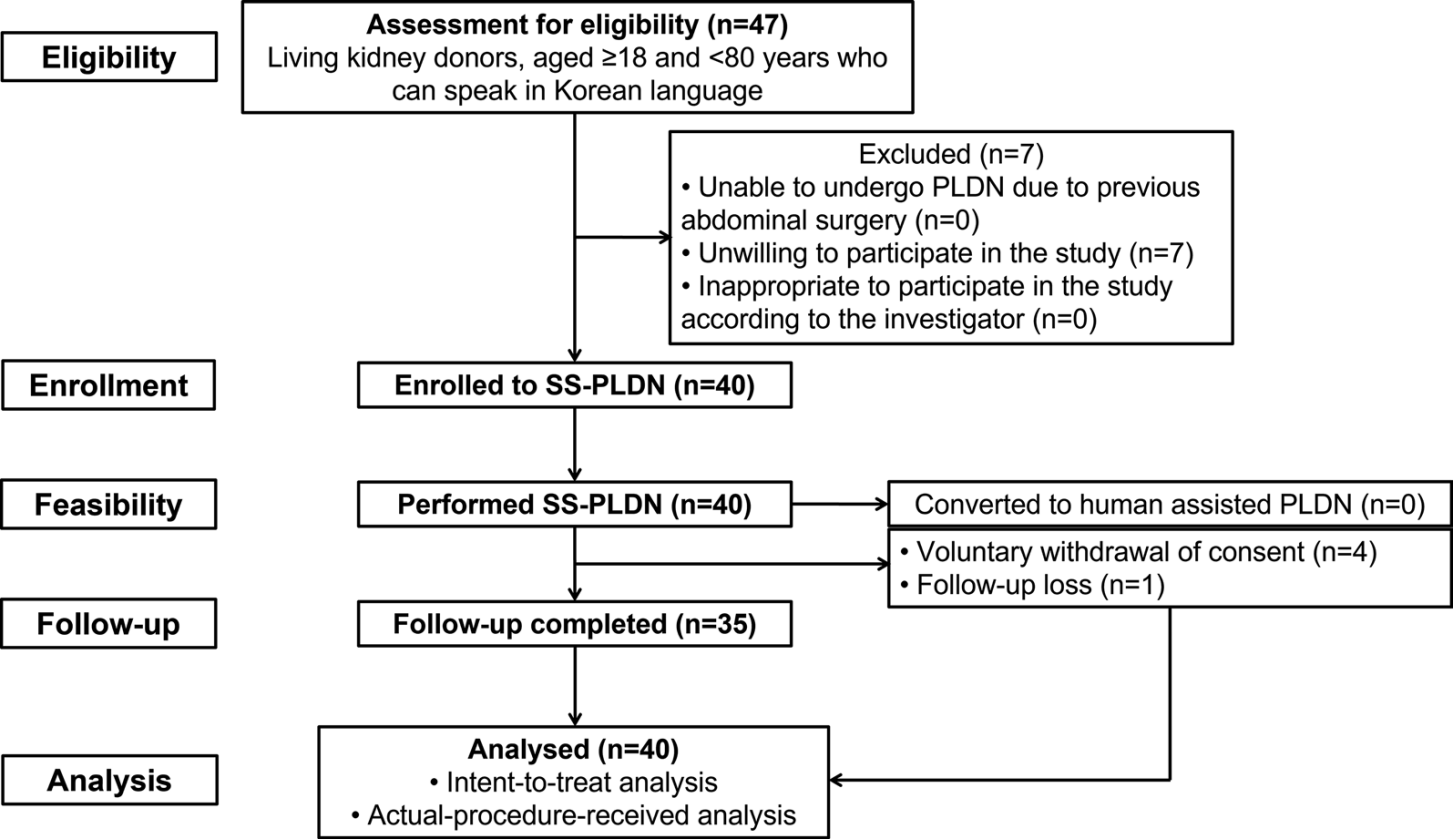
Flow diagram of IDEAL stage 2a study

Of the 47 potential candidates from November 2018 to August 2019, 40 were enrolled in the clinical trial and seven excluded as they declined consent for participation. One patient dropped out shortly after the surgery owing to voluntary withdrawal of consent. Subsequently, there were four drop outs at postoperative day 90 because of voluntary withdrawal of consent in three and failure to visit in one patient, respectively (Fig. 2). The baseline characteristics of enrolled patients are outlined in Table 1.

**Table 1:** Baseline characteristics

Characteristics	
Age, year	50.4 ± 10.1
Sex	
Male	15 (37.5)
Female	25 (62.5)
Height, cm	162.6 ± 9.5
Body weight, kg	66.5 ± 11.5
Body mass index, kg/m^2^	25.0 ± 2.4
Diabetes mellitus	1 (2.5)
Hypertension	6 (15)
History of abdominal surgery	13 (32.5)
Laterality	
Right	16 (40)
Left	24 (60)
No. artery	
1	32 (80)
2	7 (17.5)
3	1 (2.5)
No. vein	
1	31 (77.5)
2	7 (17.5)
3	1 (2.5)
4	1 (2.5)
Preoperative hemoglobin, g/dL	13.6 ± 1.6
Preoperative GFR, mL/min/1.73 m^2^	99.7 ± 10.8

GFR, glomerular filtration rate

### Primary outcomes

The solo-surgery operation time of all cases was 120 min or less and there was no occasion of any difficulty that required conversion to the human assisted PLDN. Accordingly, the feasibility of SS-PLDN was 100% (40/40).

Intra- and postoperative complications are outlined in Tables 2 and 3, respectively. Ten patients (25%) experienced a total of 14 intraoperative errors (Table 2). The most common intraoperative complication was spleen injury, followed by liver and bowel injury. According to the definition of Satava classification, these grade I errors did not require any special action. There were a total of three intraoperative complications classified as the Satava classification grade II, two of which were due to the incomplete stapling of endoscopic stapler, and the third was due to the electro-coagulator during skin incision. There were no intraoperative complications associated with the corresponding to grade III of the Satava classification. Thirty-four patients (85%) experienced a total of 78 postoperative complications (Table 3). The most common postoperative complication was nausea/vomiting and followed by aspartate aminotransferase/alanine aminotransferase elevation. There were no postoperative complications under the influence of solo-surgery and no postoperative complications that would be classified as the Clavien-Dindo classification grade III or higher.

**Table 2:** Intraoperative complications

Intraoperative complications	
Grade I	
Spleen (capsular or parenchymal) injury	4
Liver (capsular or parenchymal) injury	2
Bowel injury	2
Adrenal gland injury	1
Dislocation of clip from vessels	1
Mesentery injury	1
Grade II	
Incomplete stapling of endoscopic stapler	2
Bowel injury needing repair	1

**Table 3:** Postoperative complications

Postoperative complications	
Grade I	
Nausea/vomiting	21
Aspartate aminotransferase/alanine aminotransferase elevation	9
Extremity musculoskeletal pain or numbness	7
Headache	6
Lower urinary tract symptoms	4
Urticaria or contact dermatitis	4
Atypical chest pain (negative cardiac workup)	3
Cough	3
Neck/shoulder/back pain	3
Wound infection confined epidermis	3
Ileus needing enema or diet delaying	2
Abdominal pain	1
Atelectasis	1
Diarrhea	1
Generalized edema	1
Oliguria	1
Orchialgia	1
Grade II	
Hypertension	2
Uveitis	2
Desaturation	1
Sleeping tendency	1
Transfusion	1

### Secondary outcomes

The operative and convalescence parameters are outlined in Table 4. Mean total and solo-surgery operation time were 175.6 ± 27.8 and 97.8 ± 15.0 min, respectively. Mean warm ischemic time and estimated blood loss were 4.2 ± 1.8 min and 112.9 ± 66.6 mL, respectively.

**Table 4:** Operative and convalescence parameters

Operative parameters	
Total operation time, min	175.6 ± 27.8
Solo-surgery operation time, min	97.8 ± 15.0
Warm ischemic time, min	4.2 ± 1.8
Estimated blood loss, mL	112.9 ± 66.6

Convalescence parameters	
Visual analog scale on postoperative day 1	4.1 ± 1.6
Visual analog scale at discharge	1.4 ± 1.0
Interval to return to regular diet, days	3.0 ± 0.9
Hospital stay, days	5 ± 1.1
Hemoglobin on postoperative day 0, g/dL	11.8 ± 1.3
GFR on postoperative day 0, mL/min/1.73 m^2^	79.7 ± 13.3
Hemoglobin on postoperative day 30, g/dL	13.2 ± 1.2
GFR on postoperative day 30, mL/min/1.73 m^2^	71.7 ± 14.7
Hemoglobin on postoperative day 90, g/dL	13.4 ± 1.2
GFR on postoperative day 90, mL/min/1.73 m^2^	68.1 ± 11.4

GFR, glomerular filtration rate

## Discussion

Since its introduction, laparoscopic surgery has demonstrated several advantages compared to open surgery, in terms of postoperative pain, recovery time, fewer wounds and the need for less manpower during surgery. Lesser invasive techniques such as reduced ports or single port laparoscopic surgery are currently being studied [11]. In keeping with these changes, solo-surgery has been introduced to reduce the number of required assistants. Recently, the development of surgical instruments has facilitated the use of solo-surgery [12]. The basic instruments which enabled surgeons to perform the solo-surgery were camera holders. Depending on how the camera holder is operated, it is divided into active and passive. An active camera holder is operated by an electric motor and uses head movements, voice control, finger or foot-operated switches as a user interface for operating the camera holder. Representative active camera holders include AESOP robots (Automated Endoscopic System for Optimal Positioning robots; Computer Motion, Coletta, CA, USA), EndoAssist (Armstrong Healthcare Ltd, High Wycombe, UK), and ViKY (Endocontrol Medical, Grenoble, France). Passive camera holders are manually controlled and subdivided into one-handed and bimanual types. Representative one-handed passive camera holders include Unitrac (AESCULAP, Tuttlingen, Germany), Endofreeze (AESCULAP), Laparostat (CIVCO, Carolville, IA, USA), and POINT SETTER™ (Karl Storz, Tuttlingen, Germany). Representative bimanual passive camera holders include Martin arm (Gebuder Martin, Tuttlingen, Germany), Karl Storz Holding system (Karl Storz), and Assisto (GEOMED, Tuttlingen, Germany).

The characteristics of solo-surgery were derived from replacement of human assistants with these camera holders. This had several advantages. First, the solo-surgeon gets the steady field of view they require. Human assistants without enough experience of surgery could not provide a steady field of view and these incompatible views made the operator uncomfortable. Because the solo-surgeon modulated the camera holder as intended, discomfort due to unintended views did not occur. Second, since there were no human assistants, more space was available to the solo-surgeon. Using the entire space shared which used to be earlier shared with the assistants, the movement became easier and there was less room for the operation to be interrupted. Additionally, manpower allocation for human assistants was also efficient. Particularly, there was a merit that the manpower could be used more efficiently in the areas where there is a shortage of resident manpower due to the decrease in resident and support such as Canada, Taiwan, and Korea [13–15].

On the other hands, there were also some disadvantages of solo-surgery. If the surgeon is unfamiliar with the use of the camera holder, a fixed camera holder might rather interfere with the movement of the solo-surgeon. Unlike the assistant who can move cooperatively, the camera holder was fixed from the beginning to the end of the operation, so if it is fixed in the wrong position, it might interfere with the operation and may require skills in positioning the camera holder. In addition, the absence of medical personnel to cope with emergencies can be a problem. There may be concerns that surgical training for residents will be difficult with the advent of solo-surgery [1].

The most important findings of the present study include that the bimanual manipulation of passive camera holder was easy to learn and did not cause any difficulties to make SS-PLDN impossible. It may take longer because the surgeon has to perform the operation while changing the camera position. However, since there is no process of instructing the assistant to operate the camera, it can reduce unnecessary operation time.

The intraoperative complication rate in the present study was higher than that reported in patients undergoing human assisted PLDN (25% in the present study versus 2.8%–25% in human assisted PLDN) [16]. Spleen, liver, and bowel injuries can occur unintentionally during laparoscopic surgery from port placement to wound closure. These injuries can occur while replacing laparoscopic surgical instruments. To avoid injury, the assistant must move the camera to show the inside of the trocar when inserting surgical instruments. Because solo-surgery makes this impossible, unintended injury can often occur. Complete and meticulous perihilar dissection is the key step that facilitates proper stapling of endoscopic stapler [17]. A fixed camera during solo-surgery makes perihilar dissection difficult and may lead to incomplete stapling of the endoscopic stapler. To prevent this complication, it is better to perform perihilar dissection and renal pedicle ligation together with an assistant.

The postoperative complication rate was also higher than previously reported (85% in the present study versus 0%–43% in human assisted PLDN) [16]. The higher complication rates may be due to prospective recording by systematic classification methods, including minor complications [7]. Nevertheless, the postoperative complication rate in the present study was somewhat higher than to those in human assisted PLDN performed by same surgeon from 2014 to 2018 [8]. This might be attributable to checking the patient's symptom and sign every day after SS-PLDN. However, most postoperative complications were likely unrelated to solo-surgery itself.

Operative and convalescence outcomes in the present study were similar to those in human assisted PLDN performed by same surgeon from 2014 to 2018 [8]. Especially, the warm ischemia time was not longer than reported previously in patients undergoing human assisted PLDN (4.2 min in the present study versus 2.6–5.4 min in human assisted PLDN) [18, 19].

The main limitation of the present study is limited series with short follow-up. Although the present study determined the feasibility of SS-PLDN, the IDEAL stage 2b study including a randomized controlled trial would be needed to clarify the safety and potential benefits compared to human assisted PLDN [5]. Because PLDN performed on a healthy person who is not needing surgery, it has to be as safe as possible in terms of perioperative and potentially long-term negative health effects. Therefore, in the present study, the assistant was waiting in the operating room for possible emergency situations during SS-PLDN. This was an action taken in accordance with the recommendation of the Institutional Review Board. Given these shortcomings, PLDN can be a good choice as the first start of the solo-surgery. There is no change in the normal anatomy due to the disease and only normal variation, so the possibility of unexpected variations during operation is low.

## Conclusions

SS-PLDN using a passive camera holder was found to be technically feasible in this IDEAL stage 2a study. In terms of safety, it is necessary to adjust the scope of surgery performed alone.

## Data Availability

The datasets used and/or analysed during the current study are not openly available due to human data but are available from the corresponding author on reasonable request.
